# Authors’ reply

**Published:** 2011

**Authors:** Anjum Darakshan, Abadan K Amitava

**Affiliations:** Institute of Ophthalmology, JN Medical College, Aligarh Muslim University, Aligarh - 202 001, Uttar Pradesh, India

Dear Editor,

We are grateful for the interest taken by Gupta *et al*.[1] in our article[2] and for the thoughtful and pertinent issues raised by them.

We noticed the reference to the article by Solares *et al*.[3] in Tonelli’s paper,[4] but since we could not access the original paper, we refrained from citing it. We have been careful to frame our sentence to say that “we could access only one comparable human study in the literature,” when referring to Mulet’s article.[5] We agree that Solares *et al*. first reported good results in 10 patients.

Although there exists the possibility of the muscle tendon slipping within its sheath, one possible reason it did not occur in our cases was because the sheath had been transfixed to the tendon due to the “safety suture:” this included a belly bite in the center and two whip stitches at the edges near the insertion with Vicryl 6-0. As for the risk of the drop rolling off posteriorly from the sclera, a matter addressed by increasing the viscosity of the sealant by Mulet,[5] we may not have been able to be clear in our description of the precise technique in our paper.[2] While we placed a drop of the sealant onto a metal spoon, it was only lifted by the 25-gauge cannula. This thus permitted insufficient sealant onto the cannula, such that in none of the cases did any drop (or a part of it) trickle posteriorly, and nor did it appear enough to lead to a situation of further extensive and more posterior adhesion (on account of capillarity) mimicking a Faden operation. Nevertheless, the authors are correct to raise such a possibility.

In none of the 10 cases in our series did the attachment fail in the first instance itself, and so neither a reapplication nor a switch to sutural recession surgery was performed.

We stand corrected: the [Table 1] data are correct, and the text in results should read, “There were six exotropes and four esotropes.”

**Table 1:** The preoperative type and amount of strabismus and postoperative outcomes (at 4- to 6-week follow-up) in terms of fusion, stereopsis, and strabismus of the 10 cases in this series

BCVA		Preop. strabismus (PD)	Bilateral rectus recessions	Postop. strabismus (PD)	Bagolini striated glasses	Stereoacuity (arc-sec) on the Titmus fly test
RE	LE					
Plano 20/20	Plano 20/20	X[T]−45	LR 8 mm	Orthotropia	Fusion	60
Plano 20/20	Plano 20/20	ET+35	MR 5 mm	EP +12	Fusion	50
−3.0 20/20	−3.0 20/20	XT−55	LR 9.5 mm	X[T]−16	Fusion	50
+0.5+0.5 × 180 20/20	+1.5×180 20/20	XT−50	LR 9 mm	Orthotropia	Fusion	40
−0.5−2.5 × 90 20/20	−6.0−1.0 × 140 20/20	XT−45	LR 8.0 mm	XT (with DVD)−8	Intermittent diplopia/supp.	absent
+2.0 20/30	+3+2 × 120 20/80	Left ET+55	MR 6.5 mm	Left ET+10	Fusion	140
Plano 20/20	Plano 20/20	XT−35	LR 7.5 mm	XP−25 PD	Fusion	40
Plano 20/20	Plano 20/20	ET+50	MR 6 mm	Orthotropia	fusion	400
Plano 20/20	Plano 20/20	ET+35	MR 5 mm	ET6	Fusion	100
Plano 20/20	Plano 20/20	XT−45	LR 8 mm	Orthotropia	Fusion	40

Surgical success would mean either of the following: demonstration of fusion/any stereopsis, or orthotropia, or conversion to a phoria or intermittent tropia, or strabismus to within 10 prism diopters (PD) of orthotropia, BCVA: best corrected visual acuity, X[T]: intermittent exotropia, LR: lateral recti, ET: esotropia, MR: medial recti, EP: esophoria, XT: exotropia, DVD: dissociated vertical deviation, XP: exophoria

In all the 10 cases, the safety suture was removed 4-6 h after surgery. It was cut flush with the surface of the conjunctiva after gently pushing the latter back with spring scissors. We feel that it is highly unlikely that the remnant of the safety suture (Vicyrl 6-0) would ever be able to act as a hang-back recession in the event of slippage of a recessed muscle, since the two ends of the suture were merely passed through the insertion stump of the muscle without any anchorage. In an event of a slipped muscle, the suture would simply slip through the muscle stump and not prevent slippage.

It is not proper to compare time between an animal experimental study and a human study. By its very nature, a human study will involve a level of care and caution far greater than in an experimental environment.[6] It is clear from our study that the glue worked within 45 s: the rest of the time was spent in ensuring a dry and bloodless field to apply the glue. Even the authors have stated in the abstract that the using cyanoacrylate was faster by an average of 3.85 min for the first operations only and not for reoperations.[6] We feel that with greater use and confidence, recessions with cyanoacrylate would become both easier and quicker.

We did not report on the efficacy of the study in terms of amount of strabismus corrected since in a paired design as ours, wherein one eye of each patient was randomly allocated to be reattached with cyanoacrylate while the other was conventionally recessed using sutures, we would be unable to compare the efficacy between adhesive and sutures. However, we understand the desire for the reader to know the outcome of this series and are thus providing a table. In the present study: 9 of the 10 cases demonstrated fusion postoperatively. Of these nine, six subjects had ≤ 60 arc-sec stereoacuity, while there was one case each of 100, 140 and 400 arc-secs (range: 40-400 arc-secs). One case that did not show any stereopsis and responded with either suppression or occasionally a diplopia response on Bagolini striated glasses had a residual exotropia (XT) of 8 prism diopters (PD) with a bilateral dissociated vertical deviation (DVD). In terms of postoperative strabismus, four patients had orthotropia, two were phoric, one had converted to intermittent exotropia, two had esotropia (ET) ≤ 10 PD, while one had a manifest XT (-8 PD) (with bilateral DVD). Ocular movements were full and free in all directions. Cosmetically, all the 10 cases had a satisfactory outcome.

We are only too aware that the ideal situation would have been to have an independent assessor masked to the allocation, but due to logistic constraints we could not carry this out. Although postoperative biomicroscopy was carried out, indirect ophthalmoscopy was not. Interestingly, we recently made an effort to recall all our cases, 9 months after the last patient was operated upon. Six reported back, while we traced one to her home. One patient, from afar, declined to come, but reported satisfactory alignment and no untoward effect on his eyes. Two could not be contacted by phone/post. The eyes of these seven patients were white and quiet, and none showed any abnormality on biomicroscopy and on indirect ophthalmoscopy. In our small study, we found no postoperative complications, and have reported it as such.

## References

[1] Gupta V, Gupta P, Gupta R (2011). Isoamylcyanoacrylate for sutureless horizontal rectus recession surgery. Indian J Ophthalmol.

[2] Darakshan A, Amitava AK (2010). Cut and paste: A novel method of re-attaching rectus muscles with cyanoacrylate during recessions in strabismus. Indian J Ophthalmol.

[3] Villaseñor-Solares J, Aguirre-Aquino BI (1998). Adhesives tisulars use in surgery of strabism (sic). Rev Bras Oftal.

[4] Tonelli E, de Almeida HC, Bambirra EA (2004). Tissue adhesives for a sutureless fadenoperation: An experimental study in a rabbit model. Invest Ophthalmol Vis Sci.

[5] Mulet ME, Aliσ JL, Mahiques MM, Mahiques MM, Martνn JM (2006). Adal-1 bioadhesive for sutureless recession muscle surgery: A clinical trial. Br J Ophthalmol.

[6] de Alba Campomanes AG, Lim AK, Fredrick DR (2009). Cyanoacrylate adhesive use in primary operation and reoperation in rabbit eye muscle surgery. J AAPOS.

